# Necrotizing enterocolitis in the preterm: newborns medical and nutritional Management in a Single-Center Study

**DOI:** 10.1186/s13052-021-01180-8

**Published:** 2021-11-14

**Authors:** Giovanni Savarino, Maurizio Carta, Marcello Cimador, Antonio Corsello, Mario Giuffrè, Ingrid Anne Mandy Schierz, Gregorio Serra, Giovanni Corsello

**Affiliations:** 1 Department of Health Promotion, Mother and Child Care, Internal Medicine and Medical Specialties “G. D’Alessandro”, University Hospital “P. Giaccone”, Palermo, Italy; 2 University of Milan, Milan, Italy

**Keywords:** Necrotizing enterocolitis, NEC, Pediatric nutrition, Neonatology, Pediatric surgery, Enteral formulas, Pediatric gastroenterology, Very low birth weight infants, Preterm newborns

## Abstract

Necrotizing enterocolitis (NEC) is a typical disorder of preterm newborns, with a high mortality and morbidity rate. The therapeutic and nutritional management of disease depends on several factors. Its prognosis is linked, in addition to the severity of the disease and the need for surgery, to a correct enteral feeding in these patients. This study aims to identify the clinical characteristics of 18 patients with NEC, evaluating the different therapeutic paths undertaken, the type of formula used and the survival rate of this population. Average time of enteral nutrition before the NEC onset was 11,3 ± 11,6 days, with an average fasting period since the onset of 24 ± 18.9 days. 77.8% of patients received surgery and resumed enteral nutrition 17.7 ± 17.9 days after the intervention. The overall survival rate of our cohort was 55.5%. More prospective studies are needed to evaluate the long-term outcomes of survived children with NEC.

## Introduction

Necrotizing enterocolitis (NEC) is a critical disease typical of premature infants. Although its incidence varies among different neonatal intensive care units, the average prevalence among very low birth weight infants (VLBW), defined as those with a first recorded weight < 1500 g, is 7% [1, 2]. The intestinal barrier immaturity and a gut microbiota dysbiosis could probably contribute to the intestinal inflammation and the damage observed in these patients [1, 3].

The Bell’s staging, introduced in 1978 and later modified by Kligeman and Walsh, stratifies the severity of this pathology and guide the treatment approach [4]. In Bell’s stages I and II, corresponding to cases of just suspected or mild to moderate NEC, therapy consists of a broad-spectrum antibiotic therapy and parenteral nutrition, with a contemporary suspension of enteral nutrition for an average of 7 to14 days. In cases of worsening of symptoms or severe disease (stage III), hemodynamic instability, low platelets, CID, peritonitis or pneumoperitoneum are frequent findings. In these cases, surgery is generally indispensable [5]. Mortality rate found among NEC cases which needed surgery rises from 3 to 30% [6]. Furthermore, when a massive intestinal resection is performed, short bowel syndrome can result [5]. On a histological level, indeed, after massive resections, adaptations of the remaining bowel structure have been observed, like a lengthening of villi, a deepening of crypts and an increase in the proliferation of enterocytes. These changes increase the absorption capacity of the residual bowel [7].

The purpose of this study is to evaluate a population of patients affected by NEC, collecting their clinical data and analyzing the nutritional implications of disease.

## Material and methods

An observational prospective study was carried out through the collection of cases of NEC admitted to the Neonatology and Neonatal Intensive Care Unit of “Policlinico P. Giaccone”, Palermo between January 2015 and January 2019. We revised the medical records of these infants; data were extrapolated with a standardized table. The main outcome of this study was to evaluate the survival rate, distribution and characteristics of NEC patients in a single cohort, evaluating different approaches utilized and observing their possible clinical outcomes.

Clinical data that have been collected were clinical condition and age at diagnosis, progression of disease, therapeutic nutritional and surgical management. For each patient a multidisciplinary evaluation has been carried out through joint visits, with the presence of pediatrician, surgeon, parents. Categorical variables were synthesized with frequencies, percentages and statistical distribution.

The study was conducted according to the guidelines of the Declaration of Helsinki, and it has been approved by the ethical committee of the center involved.

## Results

**Fig. 1:**
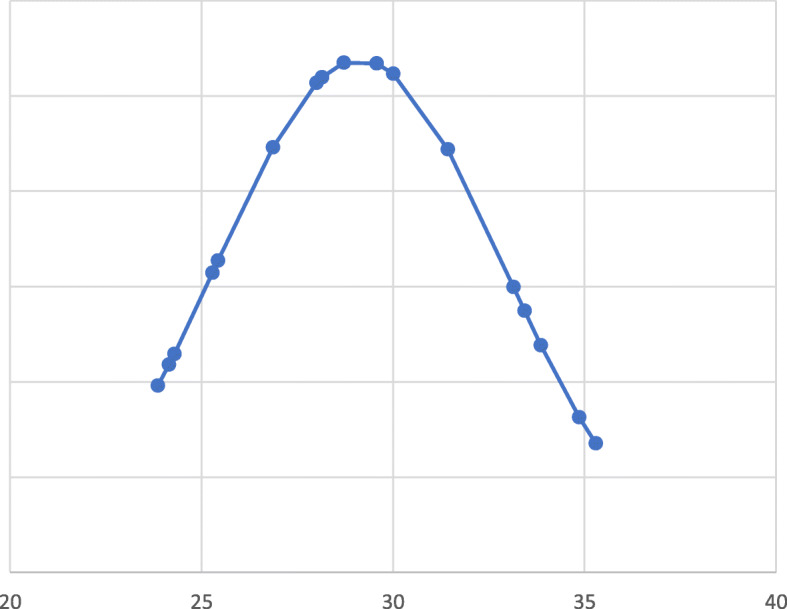
Patients for gestational age at birth (weeks) distribution

**Fig. 2:**
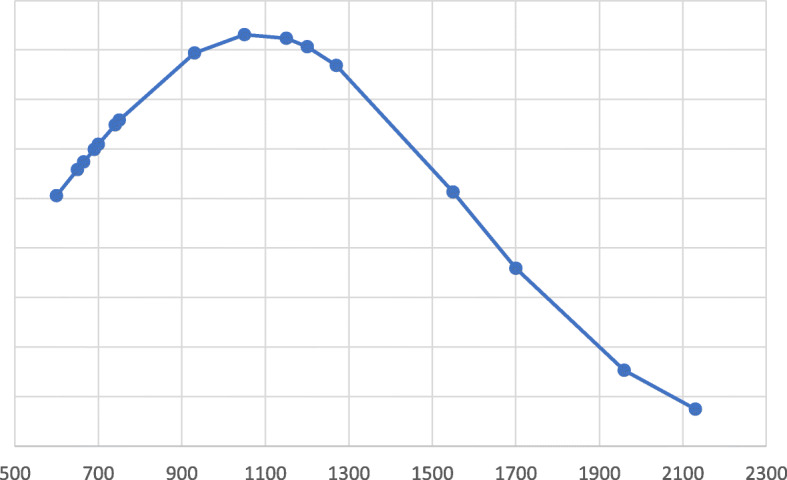
Born weight distribution (g)

In total, 18 infants were included in the study: 8 males and 10 females (Table 1). The gestational age (GE) at birth was between 23 + 6 week and 35 + 2 week, with an average of 29 + 1. 13 infants had GE < 32 w, 3 had GE between 32 and 33 w and 6 days, 2 were late preterm (GE between 34 and 36 w and 6 days). The smaller infant weighed 600 g, while the bigger one weighed 2130 g. The average weight found at birth was 1078 ± 478,96 g. 4 infants were low birth weight (LBW) (< 2500 g), 4 were very low birth weight (VLBW) (< 1500 g), 10 were extremely low birth weight (ELBW) (< 1000 g). All the infants in our population were preterm, some had specific comorbidities; in 2 genetic pathologies were found, in 5 a patent ductus arteriosus was found. In 1 newborn a hypertrophic heart disease was diagnosed. 3 newborns were from bigeminal pregnancies, 15 from single ones. Regarding severity of NEC, 14 infants were on stage III of Bell, 4 on stage II and no one was on stage I. 8 newborns died during the hospitalization, of which 7 were on stage III and 1 on stage II. The survival rate of our population was 55.5%. Figures 1 and 2 describe the distribution of cases for gestational age and weight at birth.

**Table 1:** Baseline patients’ characteristics

Newborns	18
Sex ratio M/F	0,8
Gestational age (Week)	29 + 1 [I.C. 23^+ 6^–35^+ 2^]
Weight (g)	1078 ± 478,9
NEC II	4
NEC III	14
Deaths	8

### Enteral nutrition

**Fig. 3:**
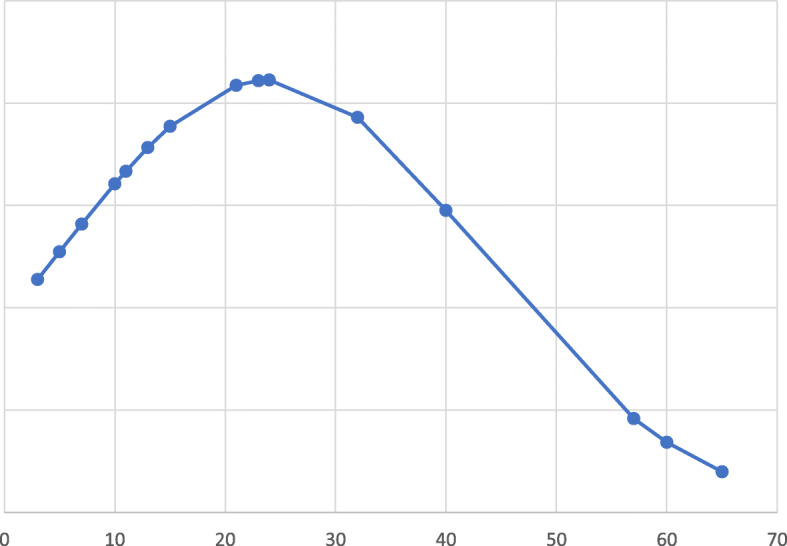
Distribution enteral fasting days after NEC onset

In our case series, enteral nutrition started on average after 6.7 ± 9.6 days of life. This delay in starting nutrition could be related to prematurity. Considering the type of milk administered before NEC, 5 groups of newborns were identified: 2 newborns were fed with breast milk and hydrolyzed formula, 5 with breast milk and preterm formula, 2 with preterm formula and 8 with breast milk only; 1 newborn developed NEC before the initiation of enteral feeding. In the 17 newborns who developed NEC after the beginning of enteral feeding, it was stopped for food intolerance after 15.5 ± 13 days of life. Infants of this case series performed enteral nutrition for 11,3 ± 11,6 days before the NEC onset. Considering the interval between disease onset and enteral reintroduction, an average fasting period of 24 ± 18.9 days was performed. Figure 3 describes the days distribution for the enteral fasting after NEC onset.

The time to reach a full enteral feeding was evaluated by measuring the interval between the day of enteral reintroduction and the day of parenteral stop. 8 infants were excluded (7 for an early exitus and 1 for lack of clinical data).

Table 2 sums up types of milk administered during the enteral feeding reintroduction phase.

**Table 2:** Type of enteral nutrition reintroduced during the healing phase

Type of formula	Newborns
Hydrolyzed formula	4
Breast milk only	5
Breast milk and hydrolyzed formula	3
Breast milk and preterm formula (0)	2
Not refed due to early death	4

### Parenteral nutrition

In our series, due to the prematurity, many infants started parenteral nutrition before NEC onset. It should be noted that in the event of death, the day of exitus was considered the day of parenteral suspension. On average, infants in our population practiced parenteral nutrition for 50 ± 28 days. Regarding the composition of the parenteral formula administered,12 newborns received olive oil lipid emulsions, 5 received soy-bean lipid emulsions and 1 received parenteral with lipid-free formula.

### Surgery

4 patients did not have surgery (22.2%) and 14 had surgery (77.8%). Surgery was performed on average after 32.5 ± 27.1 days of life. Among operated infants, 5 had an intestinal resection with ileostomy placement (27.8%), 4 had only ileostomy packaging (22.2%), 1 had a colostomy, 2 had neither resection nor ileostomy. 2 patients were treated exclusively with an abdominal drainage. Within the group of operated infants, the reintroduction of enteral nutrition occurred on average after 17.7 ± 17.9 days from surgery.

### Use of supplements and medications

Iron, D and K vitamins supplementation is essential in preterm newborns [8] Our population, in addition to the inherent risk of prematurity, was more exposed to a vitamin malabsorption and to trace elements lack. In addition to this, newborns who practice parenteral nutrition for long periods may develop carnitine deficiency [8]. We evaluated vitamin supplementation in our population: 2 newborns received carnitine, 4 oral iron, 1 injected trace elements and 9 received oral multivitamins.

We evaluated the administration of antacids and ursodeoxycholic acid (UDCA) in our population, trying to identify an indirect parameter that would give us an overview on main gastroenterological sequences of NEC (ex. gastric hypersecretion and liver disease). Many neonates with NEC, especially those who had jejunostomies, showed an increased gastric secretion that may require antacids [5]. In our population 6 infants received antiacids. Furthermore, infants with NEC, due to a prolonged parenteral nutrition, show higher risk of developing liver disease and cholestasis. These conditions need for nutritional modifications and UDCA supplementation. In our population 4 infants received UDCA.

### Signs and symptoms of NEC

Many clinical and radiological signs were evaluated from the onset of NEC. Principal signs of food intolerance which brought to the interruption of enteral feeding were abdominal distension (83.2%), stagnation (33.3%) and intestinal bleeding (27.7%) (Table 3). Most frequent systemic signs were the increase of inflammation indices (55.5%) and thrombocytopenia (27.8%) (Table 4). Radiological findings observed were pneumoperitoneum (38.9%), hydro-aerial levels (38.9%) and failure to gasify the rectum (33.3%) (Table 5). Portal pneumatosis and aerobilia were less frequent radiological signs.

**Table 3:** Intestinal signs of NEC

	n	%
Distended abdomen	15	83.2
Biliary gastric stagnations	7	38.9
Hematochezia	4	22.2
Melena	1	5.6
Closed alvo	1	5, 6
Rectorrhagia	1	5.6

**Table 4:** Systemic signs of NEC

	n	%
Increase of inflammation indices	10	55.5
Thrombocytopenia	5	27.8
Desaturation	2	11.1
Bradycardia	2	11.1
Apnea	1	5.6
Neutropenia	1	5.6
Skin discoloration	1	5.6
Anemia	1	5.6
Temperature	1	5.6
Methemoglobin	1	5.6
Flecked skin	1	5.6
Hyponatremia	1	5.6
Metabolic acidosis	1	5.6

**Table 5:** Radiological sign of NEC

	n	%
Pneumoperitoneum	7	38.9
Air fluid levels	7	38.9
Intestinal distension	7	38.9
Failure to gasification straight	6	33.3
Portal pneumatosis	1	5.6
Aerobilia	1	5.6

## Discussion

Main problems in the prevention of NEC concern the introduction of enteral nutrition and the type of administered formula. 10 newborns started enteral feeding in the first 2 days of life, but on average enteral nutrition was started 6.7 days after birth. This variability could be referred to the heterogeneity of gestational ages. Recent evidences state that early enteral nutrition, despite not being a protective factor, does not increase the incidence of NEC, but allows a faster growth and reduces the risk of sepsis and food intolerance [9, 10]. Concerning the type of formula introduced, breast milk has been shown as a protective factor [11]. The incidence of NEC in breast-only infants is indeed 6–10 times lower than an adapted formula exclusive feeding [12]. Several non-nutritious components of breast milk contribute to the immune function of the gastrointestinal tract and increase the integrity of the mucous membrane [9, 10]. Some of these components are IgA, growth factors and polyunsaturated fatty acids. The beneficial effects of human milk are also linked to the presence of oligosaccharides capable of stimulating the growth of a healthy bacterial flora [13–17].

In our case series, the absence of infants with stage I NEC may be linked to a failure of early diagnosis, due to the birth in other hospitals. The development of specific biomarkers could help in the differential diagnosis. The high prevalence of NEC at stage III of our case studies (78%) it is probably linked to the presence of a Pediatric Surgery Ward in the same departmental complex. Low birth weight and high prevalence of surgical NECs could explain the total high mortality rate in our sample (44.5%), which is higher than the mortality described in other reports [5].

### NEC medical treatment

Medical NEC therapy consists of the administration of broad-spectrum antibiotics, the fasting and the initiation of parenteral nutrition. The duration of fasting depends on clinical, laboratory and instrumental evaluation [18]. According to recent evidence, early reintroduction of enteral feeding is associated with fewer complications, shorter duration of antibiotic therapy, faster progression to nutritional goals by age, and shorter hospitalization [19, 20]. A recent review [5] suggests that in patients on stage 1 and 2, fasting must last between 7 and 14 days. Conversely, a meta-analysis reports that there are no significant differences in terms of complications if the enteral is introduced before the fifth day [19]. A prolonged enteral fasting could have multiple negative consequences:

- favors the development of deficiencies in vitamins and nutrients
- determines atrophy of the intestinal mucosa
- promotes bacterial overgrowth
- prolonged parenteral nutrition increasing its infectious and metabolic complications.

Clinicians should minimize fasting, reintroducing enteral nutrition as soon as there is a clinical improvement witnessed by the stability of vital signs, the abdominal physical examination, and the normalization of platelet count and radiologic signs The identification of biomarkers (NIRS, I-FABP, intestinal alkaline phosphatase) [21, 22], capable of reflecting the severity of NEC and intestinal recovery, could help to personalize the moment of resumption of enteral feeding, minimizing the consequences of a prolonged fasting. In addition to the correct timing for the resumption of enteral nutrition, the type of formula to be used is an important factor. The superiority of breast milk seems undisputed, but when it is not available, it is not known whether the adapted formula or the hydrolysate is preferable. In our series, 10 newborns resumed enteral nutrition with breast milk (5 exclusively, 3 associated with hydrolyzed formula, 2 associated with adapted formula), 4 resumed enteral nutrition with hydrolyzed milk, the remaining 4 did not resume enteral nutrition because they died earlier. After enteral nutrition reintroduction, it is necessary to assist the newborn until full enteral feeding is reached: this interval in our population lasted after 17.2 ± 8.2 days on average.

### Parenteral nutrition

Neonates with NEC should start early parenteral nutrition with an adequate dose of amino acids (3.5–4 g/kg/day), in order to maintain a positive nitrogen balance, an improvement of weight growth and allowing the repair of damaged tissues [1, 23–25]. Parenteral nutrition can be discontinued when enteral nutrition is sufficient to meet the nutritional needs. Prolonged parenteral nutrition is associated with higher risk of infections and metabolic problems, such as dyslipidemia and liver disease. The duration of parenteral nutrition in our series is particularly long if compared to other studies in the literature, but this data could be due to the prematurity and severity of NEC in our newborns. In our series, parenteral nutrition was started on average on the second day of life and practiced for an interval of 50 ± 28 days. In our case series, 12 newborns received olive oil lipid emulsions; 5 received soybean lipid emulsions; finally, 1 newborn practiced parenteral nutrition without lipids.

Main metabolic complications of the parenteral nutrition are liver disease and intestinal failure (IFALD) [26]. These complications incidence could be modified by reducing the duration of parenteral nutrition, preferring cyclic infusions and using suitable lipid mixtures. Several factors should be taken into consideration when choosing the lipid emulsion to use for parenteral: the content of essential fatty acids, the ratio of omega-6 and omega-3 and the amount of α-tocopherol and phytosterols. Soy-bean lipid emulsions have an omega-6: omega-3 ratio of 7:1, while the ideal ratio should be 4: 1 [27, 28]. In addition, they have a high content of phytosterols which cause liver inflammation and cholestasis [29, 30]. Fish oil lipid emulsions are mainly composed of omega-3 and contain low amounts of omega-6 [31]. Therefore, fish oil emulsions are approved as IFALD therapy, but not for ordinary pediatric parenteral nutrition due to the risk of deficiency of essential omega-6 fatty acids.

Recently, new lipid mixtures called Smoflipid have been developed. They are composed by soybean oil (30%), coconut oil (30%), olive oil (25%) and fish oil (15%).

In children with cholestatic jaundice, there is an improvement in liver function after switching from soy-bean lipid emulsions to Smoflipid [32]. Smoflipid has a positive impact on liver enzymes thanks to the low content of phytosterols and to the high content of vitamin E. Its use determines a reduction in the lipid peroxidation and an optimization of the ratio between PUFA omega-3: omega-6 which creates a minor pro-inflammatory insult [33].

### Surgical NEC treatment

In cases of NEC complicated by intestinal perforation, surgery is required. In our sample, 14 newborns underwent surgery. Among the 14 operated infants, 5 underwent intestinal resection with enterostomy packaging, 5 had enterostomy packaging only, 2 underwent a laparotomy without resection or enterostomy, and finally 2 were treated exclusively with the placement of an abdominal drainage. After extensive bowel resections intestinal failure can develop [34]. This condition can be overcome thanks to an intestinal adaptation that allows the achievement of enteral autonomy [35]. The length of the residual intestine and the type of nutrition influence intestinal adaptation. Post-operative nutrition strategies that aim to enhance intestinal adaptation are a cornerstone of treatment.

### Enteral nutrition in surgical NEC

**Fig. 4:**
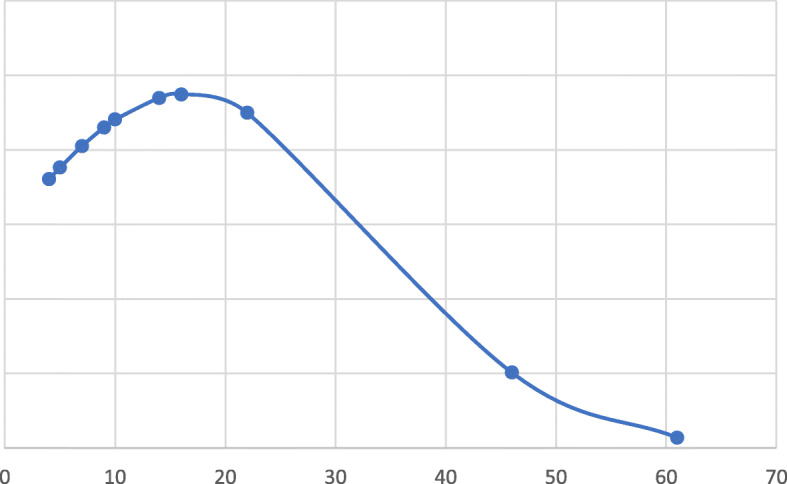
Distribution of days between surgery and enteral re-feeding

In our series, patients who received surgery resumed enteral nutrition 17.7 ± 17.9 days after the intervention. Figure 4 describe the distribution of different intervals of enteral refeeding in patients who underwent surgery. 7 newborns were refed with breast milk (4 exclusively, 3 together with the hydrolyzed formula), while 4 took the enteral exclusively with the hydrolyzed formula. Breast milk offers numerous benefits on the intestinal adaptation, thanks to the high content of growth factors [36]. Breast milk contains oligosaccharides such as 2-fucosyllactose (2-FL) which positively regulate the intestinal microbiome by stimulating the proliferation of enterocytes [37].

### Parenteral nutrition in surgical NEC

In patients of our series with NEC at stage III (*n* = 14), parenteral was practiced for 53.4 ± 29 days: 11/14 received olive oil lipid emulsions, 2/14 soybean lipid emulsions, finally 1/14 received lipid-free emulsions. Surgical NEC typically requires prolonged use of parenteral nutrition (> 21 days) [5]. Patients with fewer residual bowels require more days of parenteral nutrition [38–42]. Parenteral support after 28 days of surgery is associated with an increase in mortality at 1 year [43]. In clinical practice, intestinal failure can be indirectly measured by the length of parenteral nutrition required for normal or recovery growth [44].

### Use of supplements

Ostomies result in losses of mineral salts and micronutrients, especially zinc. Intestinal resections alter the absorption of certain nutrients, depending on the intestinal tract resected. In case of jejunostomy, the absorption of iron and zinc can be impaired. In ileal resections and fasting, the absorption of vitamin B12 and the resorption of biliary salts is altered. In addition, infants who practice prolonged exclusive parenteral may develop carnitine deficiency. Prolonged parenteral can cause cholestasis that reduces the resorption of fat-soluble vitamins.

## Conclusions

Our report confirm that NEC may be considered a heterogeneous and nutritional disease, which poses a series of problems, mainly related to different patients with different risk factors related to different ages, with very different surgical approaches. For this reason, each patient has specific nutritional risks, and should be evaluated by nutritionists or neonatologists with specific knowledge to assess a proper management, a correct fasting period and how to manage parenteral nutrition.

Breast milk represents the only real prevention of NEC, and it is also beneficial for newborns after surgery. It is ideal for re-nutrition both for digestibility and for the ability to stimulate intestinal trophism [19, 20, 45]. No other nutritional strategies were found useful in the prevention of NEC; even early enteral nutrition (< 96 h) or intermittent feeding, which can be considered beneficial and safe practices for low-birth weight infants and preterms, do not reduce the incidence of NEC [45–47].

The main challenge in the management of this disease is an early diagnosis, which could lead to a reduction in terms of mortality and morbidity. The identification of a specific biomarker could then allow early diagnosis or provide information on prognosis and severity.

Regarding different nutritional strategies in newborns with NEC, an excessively prolonged fasting should be avoided because it could increase the duration of parenteral nutrition and promotes intestinal atrophy. Enteral nutrition should be introduced within 7 days in stage I and II cases of NEC, and within 14 days in patients on stage III NEC [5].

Newborn with NEC have various risk factors for nutritional deficiencies: prematurity, intestinal damage, fasting, prolonged parenteral nutrition and possible presence of enterostomies [5]. A macronutrient deficiency can cause energy protein malnutrition. Deficiencies in micronutrients can interfere with the normal growth process, therefore exogenous supplementation is often required [8].

In cases of possible surgical therapy, it must be considered that it could cause malabsorption, ending up in a possible short bowel syndrome. Considering all these implications, in order to manage and limit the sequelae of the disease, the newborn with NEC needs for a periodic and frequent multidisciplinary evaluation made by a neonatologist, surgeon and nutritionist.

Our study can be considered limited by the single center approach, although this decreases the homogeneity in the management approach of disease. Another limit of this study, can be the variability in weight and gestational age of preterms.

Principal strength of this study, instead, is the number of patients for such a rare disease, which helped to identify the epidemiological characteristics of NEC patients and the different possible therapeutical management that can be performed.

More studies are needed to identify the ideal nutritional strategy for these infants and how to identify specific pathology markers. Furthermore, a prospective evaluation of neonates with NEC may be useful to evaluate the quality of life of these patients in the long-term, considering the nutritional, auxological and neurological outcomes.

## Data Availability

The datasets used and analyzed during the current study are available from the corresponding author on reasonable request.

## Abbreviations

CID: Disseminated intravasal coagulation
ELBW: Extremely low birth age
GE: Gestationalage
I-FABP: Intestinal fatty acid binding protein
IFALD: Intestinal failure-associated liver disease
NEC: Necrotizing enterocolitis
NIRS: Near infrared spectroscopy
PUFA: Polyunsaturated fatty acids
UDCA: Ursodeoxycholic acid
VLBW: Very low birth weight
2-FL: 2-fucosyllactose
